# Poly-victimisation and health risk behaviours, symptoms of mental health problems and suicidal thoughts and plans among adolescents in Vietnam

**DOI:** 10.1186/s13033-016-0099-x

**Published:** 2016-10-10

**Authors:** Minh T. H. Le, Sara Holton, Huong T. Nguyen, Rory Wolfe, Jane Fisher

**Affiliations:** 1 Jean Hailes Research Unit, School of Public Health and Preventive Medicine, Monash University, 6th Floor, the Alfred Centre, 99 Commercial Road, Melbourne, VIC 3004 Australia; 2 Faculty of Social Sciences, Behaviours and Health Education, Hanoi School of Public Health, 138 Giang Vo street, Ba Dinh district, Hanoi, Vietnam; 3 Department of Epidemiology and Preventive Medicine, School of Public Health and Preventive Medicine, Monash University, Level 6, the Alfred Centre, 99 Commercial Road, Melbourne, VIC 3004 Australia

**Keywords:** Poly-victimisation, Adolescence, Depression, Anxiety, Suicide, Vietnam

## Abstract

**Background:**

Limited evidence is available about poly-victimisation (exposure to multiple forms of victimisation) and mental health among adolescents in low and lower-middle-income countries. The aim of this study was to examine the associations between lifetime exposure to poly-victimisation, health risk behaviours, symptoms of common mental health problems and suicidal ideas in the previous year among high school students in Vietnam.

**Methods:**

Participants were high school students in rural and urban districts of Hanoi, Vietnam. The data source was an anonymously-completed structured self-report survey. Lifetime exposure to poly-victimisation was assessed using the juvenile victimisation questionnaire revised 2 (JVQ R-2); mental health symptoms by the depression, anxiety and stress scale-21 (DASS-21); involvement in health risk behaviours and previous year suicidal thoughts and plans by questions adapted from the 2013 youth risk behaviour survey. Data were collected between October, 2013 and January, 2014 and were analysed using generalised structural equation modelling.

**Results:**

In total 1616/1745 (92.6 %) eligible students provided complete data. Prior year suicidal thoughts were reported by 21.4 % (95 % CI 18.5–24.5 %) of the female respondents and 7.9 % (95 % CI 6.2–9.8 %) of the male respondents. Prior year suicidal plans were reported by 7.8 % (95 % CI 5.9–9.8 %) of the girls and 4.0 % (95 % CI 2.7–5.3 %) of the boys. Poly-victimisation was associated with increased likelihood of involvement in health risk behaviours and symptoms of common mental health problems among both sexes, which increased adolescents’ risk of having suicidal ideas in the previous year. Compared to non-victims or victims of fewer forms, poly-victims were also more likely to report suicidal thoughts and plans among both girls and boys (p < 0.05).

**Conclusions:**

Overall, the results revealed that poly-victimisation was associated with increased involvement in health risk behaviours, poorer mental health and increased risks of suicidal ideas among Vietnamese adolescents. Suicidal ideas were prevalent among the students. Interventions to assist victims of violence and prevention of violence, especially poly-victimisation, among adolescents in Vietnam is therefore important.

## Background

Mental health problems, including depression and anxiety, are among the top five leading causes for burden of disease among adolescents (people aged 10–19 years) [1]. It is estimated that at least one in ten children and adolescents worldwide are affected by mental health problems in their lifetimes [2, 3]. Among adolescents, mental and behavioural disorders accounted for more than 17 % of the total 84.3 million daily adjusted life years lost in 2012 [4]. Adolescents rank mental health problems as the most important health problems for people of their age [1]. Suicide is reported to be one of the top five leading causes of death among adolescents worldwide [3]. In 2012, about seven per 100,000 adolescents died by suicide [5].

Mental health problems and suicidal behaviours are multi-factorially determined [2]. Risk factors include family, school, neighbourhood and social circumstances as well as individual characteristics. These include experiences of adverse life events [6], parental divorce [7, 8], childhood maltreatment and neglect at home [9], bullying at school [10] and neighbourhood violence [11]. For those living in resource-constrained countries, experiences of natural disasters [12], forced displacement [13], armed conflict and war [14] and poverty [15] are additional risk factors [16]. Common, non-psychotic mental health problems and suicidal behaviours are generally found to be more prevalent among girls than boys [16, 17].

In general interpersonal violence is harmful to mental health, including among young people. Investigations of exposure to individual forms of violence including physical, emotional or sexual maltreatment among children and adolescents, have found that these are associated with an increased likelihood of health risk behaviours such as alcohol consumption, illicit drug use, tobacco smoking, and sexual activity with multiple partners. Experiences of violence are also associated with an increased risk of suicidal behaviours [18–20].

Most previous investigations of the effects of violence on mental health have been limited to individual forms of victimisation. However, this approach has been criticised for ignoring the potential interactions among different forms of victimisation and accrued experiences of multiple forms of victimisation [21, 22].

In 2005, Finkelhor et al. introduced the concept of “poly-victimisation”, which referred to the co-occurrence of multiple forms of victimisation, including physical, emotional or sexual maltreatment, neglect, robbery, theft, property vandalism, threat or assault, peer or sibling victimisation, witnessing of family or community violence, exposure to gun shooting, bombing and cyber bullying [23, 24]. The juvenile victimisation questionnaire (JVQ) was developed and validated for use as a tool to assess poly-victimisation among children and adolescents in the USA [23, 25]. The scale comprises 34 questions about different forms of victimisation. A total score can be created as the sum of these 34 questions. Since the introduction of the JVQ, it has been used in diverse settings, including in Finland [26], Spain [27], China [28, 29], Pakistan [30] and Vietnam [31]. There have, however, been variations among studies in terms of the criteria used for determination of poly-victimisation. Originally, Finkelhor et al. recommended the use of a cut-off score of four or more to define poly-victimisation when experiences in a one-year period were sought [21, 25]. When lifetime experiences were examined, these authors proposed classifying the top 10 % of the sample with the highest scores on the JVQ (indicating exposure to the most number of victimisation forms) as poly-victims [32]. This led to the use of a cut-off score of 11 or more in some studies in the USA [33], eight or more in a Spanish study [34] and five or more in a Finnish study [26]. Some subsequent studies used a cut-off score of four or more [28] or 11 or more [31], which was not based on the criterion of the top 10 % of the sample, but to allow comparison with other studies. In all studies, determination of poly-victimisation was based on the total JVQ score and not on consideration of frequency or intensity of the victimisation experiences.

Despite these variations among studies, there is growing evidence that poly-victimisation is experienced by children and adolescents in high- and upper-middle income countries [26, 28, 32] and has a more substantial adverse impact on victims’ mental health, including suicidal behaviours [21, 22, 28, 34–36], than single forms of victimisation. Specifically, when poly-victimisation is accounted for in analyses, the associations between individual forms of victimisation and mental health problems are reduced significantly; or even eliminated [35]. Most research about poly-victimisation and its association with the mental health of children and adolescents has been conducted in these countries and there is only limited evidence available about the experiences of those who live in low and lower-middle-income countries [37].

Vietnam is a densely-population lower-middle income country with more than 16 million adolescents [38]. Secondary analyses of data contributed by nationally representative samples of adolescents aged 14–19 in the Survey Assessments of Vietnamese Youth (SAVY) 1 (2004–05) and 2 (2009–10) [39] revealed that victims of severe physical violence perpetrated by family members or non-family members were more likely to have feelings of low mood or suicidal thoughts compared to non-victims [17]. Among a sample of 972 Vietnamese students aged 12–15 years, Phuong et al. [40] found that violence was associated with health risk behaviours. Witnessing physical violence between their parents was associated with an increased risk of riding a motorbike at an illegal age (less than 18); and conflict with sibling(s) was associated with a higher likelihood of drinking alcohol for adolescent girls and boys. Among adolescent boys being bullied was associated with a higher risk of suicidal thoughts. Nguyen et al’s [9] investigation of 2591 students aged 12–17 in Vietnam demonstrated a dose–response relationship between exposure to more forms of child maltreatment (including emotional, physical, sexual maltreatment and neglect) and lower self-esteem and symptoms of depression and anxiety. This study was the first in this setting to examine different forms of child maltreatment. Nevertheless, it was still limited to maltreatment; other types of victimisation including dating violence or cyber bullying were not investigated.

These studies also have methodological limitations [39–42] which affect generalisation of the results. Participants [9, 40] were recruited from public schools, which are only one of the three main types of schools (public, private and centres for continuing education) in Vietnam. Exposure and outcomes in these existing studies were assessed by study-specific questions, not standardised measures, which made it difficult to compare the findings with those reported elsewhere. Important forms of victimisation such as peer or sibling victimisation, witnessing of family or community violence, property vandalism, Internet harassment and dating violence, and adolescent mental health and suicidal thoughts and plans were not assessed. They were limited to investigating up to three forms of victimisation. Overall therefore significant evidence gaps about poly-victimisation and Vietnamese adolescents’ mental health remain.

The aim of this study was to investigate the relationships between experiences of poly-victimisation, health risk behaviours, symptoms of mental health problems, and suicidal thoughts and plans among high school students in Vietnam.

## Methods

The methods have been described in detail elsewhere [43] and are summarised here.

### Settings and study sites

The study was conducted in Hanoi—the national capital of Vietnam. Study sites were four public high schools, four private high schools and two centres for continuing education. These schools cater to young people of varied academic capabilities. Students attending centres for continuing education are often from more disadvantaged families.

### Participants

The inclusion criteria were to be a student aged 16–18 (grade 10–12), literate in Vietnamese and attending the selected schools and centres. All eligible students were invited to participate.

### Data sources

Data for the study were collected using an anonymous, self-completed 86-item questionnaire, which included both study-specific questions and standardised measures.

*Social and family circumstances* participants’ demographic characteristics, including sex (female/male), residential area (rural/urban), family compositions (both parents/single parent/none of the parents), family relationship (unhappy/happy); having a chronic disease or disability (yes/no) and school sector (public/private/centre for continuing education) were assessed using study-specific questions. Experience of adverse life events were assessed using 14 fixed-choice yes/no questions recommended by Turner and Butler [6] and included exposure to fire or natural disasters, severe traffic accidents, severe illnesses, homelessness, parental unemployment, parental incarceration and death of close friends or relatives.

*Lifetime exposure to poly-victimisation* was assessed using the juvenile victimisation questionnaire revised 2 (JVQ R-2) Youth Self-reported Screener Version [44]. The JVQ R-2 has 34 items [33], assessing lifetime exposure to property victimisation, threat, assault, child maltreatment, peer or sibling victimisation, sexual assault, witnessing of family or community violence. Three JVQ-R2 supplemental items [33] assessing lifetime exposure to violence between family members, not just the parents, and cyber victimisation were added, making a total of 37 items. The juvenile victimisation questionnaire and its revisions have been widely used to assess violence against children and adolescents [21, 26–28, 33, 45, 46]. It has been shown to be reliable, acceptable and valid for use among US children and adolescents with internal consistency Cronbach alpha of 0.80 and the test–retest agreement of 95 % (range 77–100 %) [23], but had not been validated for use among Vietnamese adolescents.

*Health risk behaviours* were assessed using six items from the Youth Risk Behaviours Survey (YRBS) 2013 [47] assessing tobacco smoking, drinking alcohol, illegal drug use, physical fighting, carrying a weapon and having two or more sexual partners. A “yes” response to each of these items was categorised as involvement in health risk behaviours and a “no” non-involvement. Similar items had been found in prior research to be meaningful and acceptable to Vietnamese adolescents aged 12–17 [48].

*Stress, anxiety and depression* symptoms were assessed using the Vietnamese validation of the depression, anxiety and stress scale-21 (DASS-21-V) [49, 50]. This scale has been formally validated in Vietnam against psychiatrist-administered structured clinical interviews and found to have acceptable internal consistency (Cronbach alpha of 0.88) and sensitivity of 79.1 % and specificity of 77.0 % [50]. It yields scores on depression, anxiety and stress subscales (range of each 0–21) and a total scale score (range 0–63). In the validation study, the total scale score was found to provide the best sensitivity and specificity and was recommended for use to detect common mental disorders among Vietnamese samples. For this study, only the total scale score was therefore used as an indicator of symptoms of mental health problems.

*Suicidal thoughts and plans in the previous year* were assessed using two fixed response yes/no questions adapted from the (YRBS) 2013 [47] which had been used in the national surveys of Vietnamese adolescents [39]: “During the past 12 months, did you ever seriously consider attempting suicide?” [47] and “During the past 12 months, did you make a plan about how you would attempt suicide?” [47].

### Procedure

Study-specific questions and standardised measures which had not yet been validated in Vietnam were translated into Vietnamese by ML. The whole questionnaire was reviewed and revised by two public health researchers bilingual in Vietnamese and English, pre-tested and then pilot-tested among a small group of 17-year-old adolescents in Vietnam. Amendments, for example, that the term ‘private parts’ refers to the genitals and/or breasts, were made to maximise clarity of the questionnaire.

For the main survey, four to six classes were selected randomly at each school, and all students in the chosen classes were invited to participate. Participation was entirely voluntary. Participants completed the survey anonymously during a normal 45-min class session without any teacher or school staff present and returned the questionnaire, whether or not it had been completed, to the researchers in sealed envelopes.

### Data management and analyses

Data were entered into a password-protected database developed in Epidata 3.1 [51]. STATA 14.0 was used for all data analyses [52].

Responses to each question in the JVQ R-2 and supplemental items were coded as yes (1) or no (0) and a total “poly-victimisation” score of the 37 items was calculated [44]. The total score (range 0–37) indicated the number of forms of victimisation, among the forms examined, each participant had experienced during their lifetime. Participants were categorised into three groups based on this score: non-victims (score of 0), victims (score of 1–10) and poly-victims (>10). The cut-off score of more than 10 was used to be consistent with a previously published paper from this dataset [31] and to allow comparison with findings from elsewhere [33].

In the first step, bivariate relationships between poly-victimisation and the total DASS-21-V score was examined using one way ANOVA tests; between poly-victimisation and involvement in health risk behaviours and past year suicidal thoughts and plans by Chi square tests. These analyses were conducted separately for female and male students.

In the next step, path analyses, using generalised structural equation modelling, were performed separately for girls and boys, to investigate the relationships between poly-victimisation and the outcomes while adjusting for socio-demographic characteristics (see Fig. 3). In these analyses, involvement in health risk behaviours, DASS-21-V total score and suicidal ideas were endogenous variables. Socio-demographic variables and poly-victimisation were exogenous. The total poly-victimisation score was used to represent poly-victimisation.

Since missing data are not accommodated in STATA 14.0 generalised structural equation models [52], the path analyses included only complete data. The significance level was set at an alpha of 0.05.

## Results

None of the schools and centres refused the invitation to participate. Of the 1745 students who were eligible and invited to participate, 120 were absent on the day of the survey, seven refused to participate and two parents did not grant permission for their child’s participation. A total of 1616 students returned a completed questionnaire, yielding a recruitment fraction of 92.6 %. Ten were excluded due to a large number of unanswered questions (more than two thirds of the questionnaire), which resulted in a sample of 1606 participants. About 45 % (729/1599) of the sample were females; half (49 %, 789/1606) lived in a rural area and the majority (88 %, 1409/1596) lived with both parents.

Experiences of interpersonal violence victimisation were prevalent with more than 94 % of the participants reporting at least one form of victimisation during their lifetime and more than 31 % reported experiencing poly-victimisation, or more than 10 forms of victimisation [31]. Adolescents who suffered from a chronic disease or disability, lived with a step parent, lived in a rural area and who reported experiences of adverse events were among those most vulnerable to poly-victimisation (see [31] for a more detailed account of prevalence and socio-demographic correlates).

The percentages of girls, boys and the total sample of all students who reported health risk behaviours are shown in Table 1. Overall, tobacco smoking and drinking alcohol were the most common among the six health risk behaviours examined. Physical fighting was also prevalent with nearly one in five students reporting being involved in a physical fight in the previous 12 months. Boys were significantly more likely than girls to behave in these ways.

**Table 1:** Prevalence of involvement in health risk behaviours and previous year suicidal thoughts and plans among the study sample

Variables	Female (N = 729)^a^		Male (N = 870)^a^		Total sample^b^	
	n	% (95 % CI)	n	% (95 % CI)	n	% (95 % CI)
*Involvement in health risk behaviours*						
Ever smoked cigarette** (N = 1.599)	101	13.9 (11.4; 16.4)	394	45.5 (42.2; 48.8)	498	31.1 (28.9; 33.4)
Ever drink alcohol** (N = 1.602)	430	59.1 (55.5; 62.6)	680	78.4 (75.7; 81.2)	1115	69.6 (67.3; 71.9)
Ever used drugs* (N = 1.602)	6	0.8 (0.2; 1.5)	25	2.9 (1.8; 4.0)	31	1.9 (1.3; 2.6)
Physical fighting in last 12 months** (N = 1.602)	92	12.6 (10.2; 15.1)	209	24.1 (21.2; 26.9)	302	18.9 (16.9; 20.8)
Carrying a weapon in last 30 days** (N = 1.599)	17	2.3 (1.2; 3.5)	64	7.4 (5.6; 9.1)	81	5.1 (4.0; 6.1)
Having had two or more sexual partners* (N = 1.533)	22	3.2 (1.9; 4.5)	50	5.9 (4.3; 7.5)	72	4.6 (3.6; 5.7)
Any health risk behaviours** (N = *1586)*	*451*	*63.2 (59.6; 66.7)*	*725*	*83.8 (81.4; 86.3)*	*1181*	*74.4 (72.3*-*76.6)*
*Suicidal thoughts and plans*						
Past year suicidal thought (N = 1.599)	156	21.4 (18.5; 24.5)	69	7.9 (6.2; 9.8)	225	14.1 (12.4–15.8)
Past year suicidal plan (N = 1.601)	57	7.8 (5.9; 9.8)	35	4.0 (2.7; 5.3)	92	5.7 (4.6–6.9)
*Any suicidal ideas (thoughts or plans) in the previous year (N* = *1599)*	*159*	*21.9 (18.9; 25.0)*	*74*	*8.5 (6.7; 10.4)*	*233*	*14.6 (12.8*–*16.3)*

*CI* Confidence interval

* p < 0.05, ** p < 0.001 in Chi square tests for comparison between females and males

^a^For each row, the total N of females and males may due to missing data

^b^Total n of females and males may not add up to total sample n due to missing data about gender

**Fig. 1:**
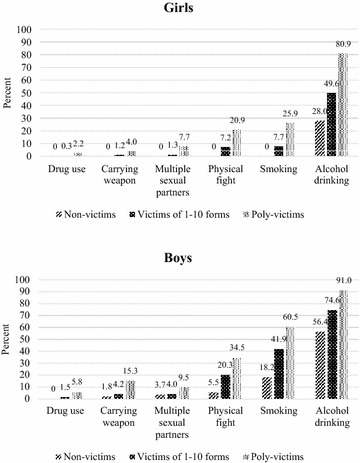
**a**, **b** Percentages of girls and boys reporting different health risk behaviours by victimisation categories among a sample of 1606 Vietnamese high school students

Among female adolescents, compared to non-victims or victims of up to ten forms of violence, poly-victims were significantly more likely to be involved in almost all of these behaviours, except for having multiple sexual partners (see Fig. 1a) (p < 0.05 for all Chi square tests, except “multiple sexual partners”). Among male adolescents, poly-victims were significantly more likely to participate in all of these behaviours (see Fig. 1b; p < 0.05 for all Chi square tests).

Students in this sample had a DASS-21-V total mean score of 15.4 ± 11.2. Girls had significantly higher mean DASS-21-V total score (17.5 ± 10.9) compared to boys (13.7 ± 11.1).

For both girls and boys, there were significant associations between poly-victimisation and more symptoms of mental health problems. Male students who were poly-victimised had significantly higher DASS-21-V total mean scores (18.9 ± 11.5) compared to victims of up to 10 forms (12.0 ± 10.0) and non-victims (6.6 ± 8.8) (p < 0.001 in ANOVA analysis). Similarly, female students who were poly-victimised had significantly higher DASS-21-V total mean scores (22.5 ± 11.4), compared to victims of up to 10 forms (15.2 ± 9.4) and non-victims (11.1 ± 11.0) (p < 0.001 in ANOVA analysis).

**Fig. 2:**
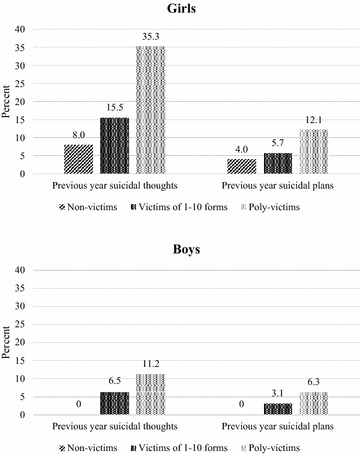
**a**, **b** Percentages of girls and boys reporting suicidal ideas in the previous year by victimisation categories among a sample of 1606 Vietnamese high school students

Suicidal ideas were prevalent among these students with 14.1 % reported having suicidal thoughts and 5.7 % having made plans for committing suicide in the prior year (see Table 1). Girls were significantly more like to have suicidal thoughts or plans in the previous year, compared to boys. Similar to health risk behaviours and mental health problems, there was significant association between poly-victimisation and suicidal ideas in the past year. For both sexes, poly-victims were much more likely than non-victims or victims to report having suicidal thoughts and plans in the last 12 months (see Fig. 2a, b) (p < 0.05 for all Chi square tests).

**Fig. 3:**
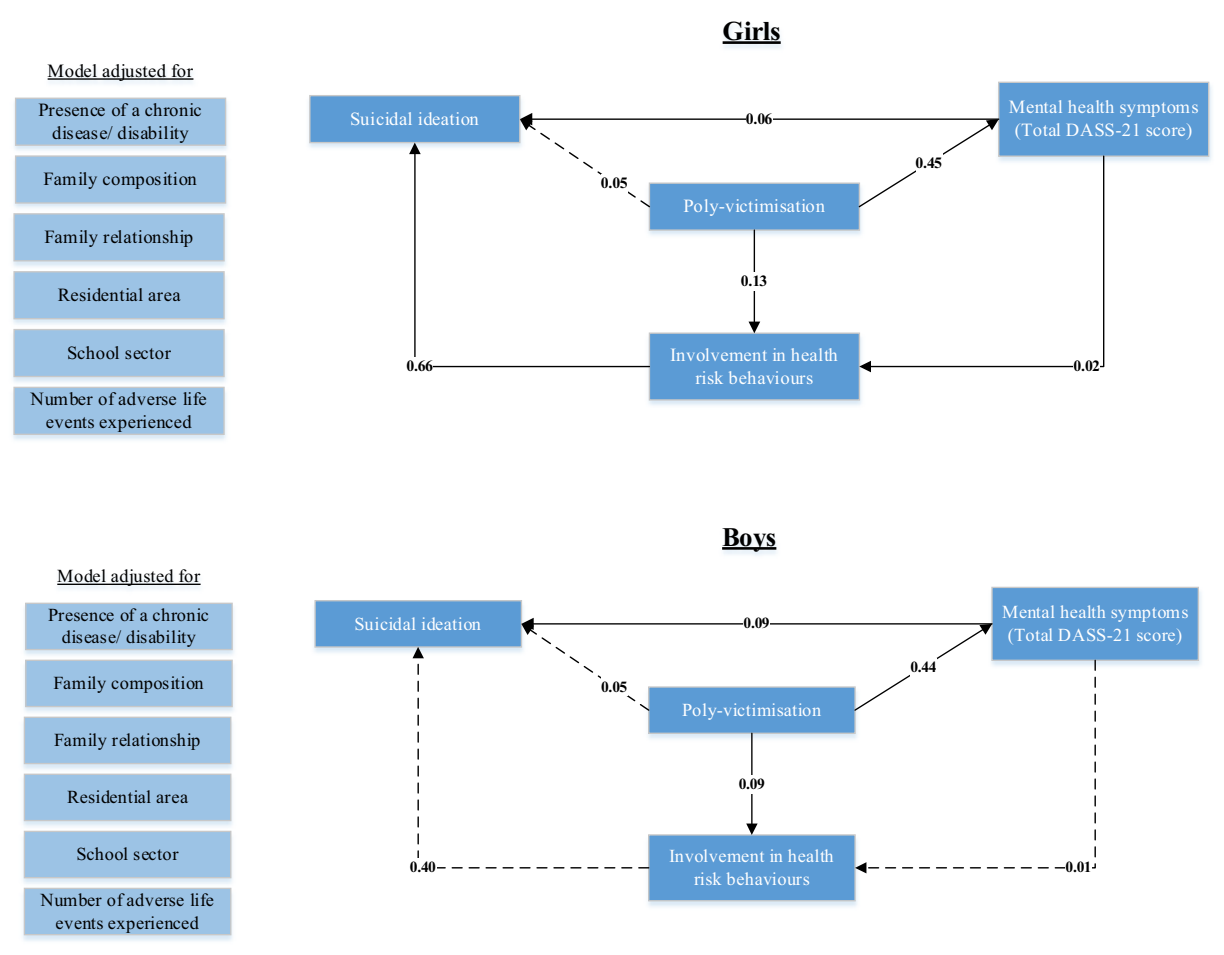
Path analysis of health risk behaviours, mental health problems, suicidal thoughts and plans: direct and indirect impacts of poly-victimisation among a sample of 1606 Vietnamese high school students. Coefficients are presented. *Solid lines* represent significant paths; *dotted lines* represent non-significant paths. Coefficients for the paths between socio-demographic variables and the three outcomes were omitted for simplicity. The error terms for the DASS-21-total score were also omitted

Results from the path analyses are presented in Fig. 3. For simplicity, coefficients for the paths between all socio-demographic factors and involvement in health risk behaviours, mental health symptoms and suicidal ideas were omitted from the figure; the error term for the DASS-21-V total score was also omitted.

Overall, path analyses revealed that for both sexes, poly-victimisation was significantly associated with increased risk of involvement in health risk behaviours and more symptoms of mental health problems (higher DASS-21-V scores). For both girls and boys, poly-victimisation was indirectly associated with an increased risk of previous year suicidal ideas via mental health symptoms (see Fig. 3).

For female students, poly-victimisation was also indirectly associated with an increased risk of suicidal thoughts and plans via involvement in health risk behaviours. These indirect impacts indicated that female adolescents who were poly-victimised were more likely to be involved in health risk behaviours and to experience mental health symptoms, both of which then increased the students’ risk of having suicidal ideas in the previous year.

For male students, the indirect association between poly-victimisation and suicidal ideas via mental health problems indicated that boys who were poly-victimised were more likely to experience symptoms of anxiety, depression or stress, which then led to suicidal thoughts and plans.

For both sexes, there was no significant direct association between poly-victimisation and suicidal thoughts and plans. Adjusted odds ratios for the direct relationships between involvement in health risk behaviours, mental health problems and suicidal thoughts and plans are presented in Table 2. For health-risk behaviours, the adjusted odds ratio suggested that girls who performed any of these behaviours were nearly two times more likely to have suicidal thoughts and plans in the previous year, compared to those who did not. For mental health problems, the adjusted odds ratio suggested that for each additional point increase in the total DASS-21-V (out of 63), there was a 6 % increase in the odds of having suicidal ideas in the previous year among girls and a 9 % increase among boys.

**Table 2:** Adjusted odds ratios for direct relationships between involvement in health risk behaviours, mental health symptoms and previous year suicidal ideas among a sample of Vietnamese high school students (results from path analysis)

Variables	Previous year suicidal ideas	
	Females	Males
	Adjusted OR (95 % CI)	Adjusted OR (95 % CI)
Involvement in health risk behaviours (yes versus no)	*1.93 (1.13; 3.30)*	1.49 (0.46; 4.82)
Mental health symptoms (DASS-21-V total scores)	*1.06 (1.04; 1.09)*	*1.09 (1.06; 1.12)*

Significant results are in italic

*OR* Odds ratio; *CI* Confidence interval; model adjusted for presence of a chronic condition or disability, family composition, family relationship, number of adverse life events experienced, residential area and school sector

## Discussion

This study is one of the very few studies from lower-middle income countries to examine the associations among poly-victimisation, health risk behaviours, mental health symptoms and suicidal thoughts and plans among adolescents and revealed that they are common and significantly related to each other.

This study was robust in including schools representing all main academic settings in Vietnam, having a high recruitment fraction, and using international standardised measures, which were formally validated and had strong psychometric properties. The use of generalised structural equation modelling allowed investigation of direct as well as indirect causal pathways between exposure to poly-victimisation and suicidal ideas. Nevertheless, we acknowledge some limitations that the schools were not randomly selected and the study was conducted in one city which might limit the generalisability of the results to high school students living in remote areas or who are out-of-school. The JVQ-R2 used to assess poly-victimisation had not undergone formal validation in Vietnam. However, extensive pre-test and pilot-test were conducted to ensure the acceptability, comprehensibility and suitability for use among Vietnamese adolescents. Information about frequency and severity of the violent experiences had not been sought. Nevertheless, previous research has shown that poly-victimisation, even when not taken into account frequency or severity, has a more detrimental impact on the victim’s mental health than chronic exposure to individual forms of victimisation [21]. There is also empirical evidence that taking into account the frequency of victimisation incidents and weighting the corresponding form of victimisation in the total number of victimisation experienced did not increase significantly the association between multiple victimisation and mental health outcomes [23]. Overall, we believe the results can be generalised with considerable confidence to adolescents attending high schools in Vietnam.

### Poly-victimisation and adolescents’ involvement in health risk behaviours

An important finding of this study was the significant associations between poly-victimisation, and adolescents’ involvement in health risk behaviours among both girls and boys. Students who were poly-victimised were more likely to participating in health risk activities, including smoking, alcohol drinking, illegal drug use, involvement in physical fights, carrying a weapon and having multiple sexual partners. It appears that the experience of poly-victimisation increases the risk of smoking which shifts girls’ behaviour from the traditional norm of non-smoking among Vietnamese women; while for boys it shifts their behaviour towards the adult male Vietnamese norm of smoking. These results are consistent with those recorded previously [29]. Among 3155 Chinese adolescents aged 12–18 years, Dong et al. [29] found significant associations between poly-victimisation, alcohol drinking and use of pornography.

### Poly-victimisation and mental health symptoms

Data from this study also reveal the apparent detrimental impacts of exposure to multiple forms of violence, crimes and abuse on adolescent mental health among both sexes in this setting. This is consistent with previous research among adolescents in Vietnam, which found that physical violence perpetrated either by a family member or a non-family-member is associated with increased risk of low mood and deliberate self-harm or suicidal behaviours [17]; and that there was a dose–response relationship between number of forms of child maltreatment and symptoms depression and anxiety and lower level of self-esteem [9]. These studies however investigated only limited forms of victimisation. It was extended in this study to include property victimisation, dating violence, peer or sibling victimisation, witnessing of domestic or community violence and Internet harassment. Overall 31.1 % (95 % CI 27.8–33.5 %) reported lifetime exposure to at least 10 forms of victimisation [43] and poly-victims had more symptoms of mental health problems.

This is similar to findings among adolescents in China [29], Finland [26], Spain [27] and the United States [11, 22]. Among Chinese adolescents, significant associations were found between poly-victimisation and symptoms of depression and anxiety [29]. Among Finnish 12–16 year-old adolescents, poly-victims were found to have increased levels of social behaviour problems, internalising as well as externalising symptoms compared to non-victims or victims of fewer forms [26].

### Poly-victimisation and suicidal thoughts and plans

In this sample, both female and male students who were poly-victimised were more likely to report having had suicidal thoughts than non-victims or victims of fewer forms of victimisation (35 % versus 8 % and nearly 16 %, respectively, among girls; and 11 % versus 0 % and 7 %, respectively among boys). They were also more likely to report having made plans for suicide in the previous year than non-victims and victims of fewer forms of victimisation (12 % versus 4 % and 6 %, respectively, among girls; and 6 % versus 0 % and 3 %, respectively among boys). There appeared to be a strong association between poly-victimisation and suicidal ideation.

However, results in path analysis revealed that when other potential confounding factors were taken into account, including the presence of a chronic disease or disability, living with a step-parent, perceived quality of family relationship, rural/urban residence, and number of adverse life events experienced, the direct association between poly-victimisation and suicidal ideation among both sexes were no longer observable. This means that for both female and male poly-victims, apart from having been subjected to multiple forms of victimisation, they also experienced other risk factors, which may have made them more vulnerable to suicidal ideation.

For both sexes, poly-victims were more likely to be experiencing symptoms of mental health problems, which then increased risk of having suicidal ideas, including when potential confounding factors were controlled. This finding of an indirect relationship between experiences of poly-victimisation and suicidal ideas are consistent with previous research in high and upper-middle-income countries, which found significant associations between poly-victimisation and mental health [11, 21, 22, 35, 53], suicidal thoughts and deliberate self-harm [28, 34] among children and adolescents. Mental health has also been shown to mediate the impact between childhood adverse events, including exposure to violence and abuse, and suicidal ideas and behaviours among South African adolescents [54].

These results can be explained by the theoretical models for suicide, which propose that suicide is the result of a multifactorial pathway and experiences of humiliation and entrapment are “central risks” [55, 56]. In this study, adolescents who are poly-victimised may have felt humiliated, trapped and finding no way to escape from their circumstances of accrued violent victimisation. They may also experience mental health problems, including anxiety or depression. All of these may have led adolescents to suicidal ideation, which they consider a solution to their circumstances [16].

For female adolescents in this sample, poly-victimisation also had a significant association with increased risks of suicidal thoughts and plans via involvement in health risk behaviours. The mediating role of health risk behaviours, such as substance use, on the relationship between exposure to violence and suicidal behaviours has also been recorded among adolescents elsewhere [57]. It is suggested that “cognitive-behavioural theory” may be applied to explain this relationship [58, 59]. This theory posits that “predisposing vulnerabilities” such as exposure to child maltreatment, may result in “maladaptive cognitive, behavioural and emotional responses to acute stressors” among adolescents [59]. When an “acute stressor” occurs, adolescents may find themselves not being able to find appropriate “adaptive solutions”, which increase their levels of distress. They may respond to this in a “distorted manner” and choose to engage in health risk behaviours, such as alcohol or substance use, which worsen their distress. Adolescents may then think about suicide as a means to “escape from a perceived intolerable internal state and hopeless life situation” [59].

### Prevalence of suicidal ideas in comparison with prior data from Vietnam and in other countries

Prevalence of past year suicidal thoughts in this sample (14.1 %) is higher than prevalence of lifetime suicidal thoughts or self-harm behaviours reported previously among nationally representative samples of adolescents in Vietnam [17] (5.3 % in SAVY 1 and 12.2 % in SAVY 2). This increasing trend in the prevalence of suicidal thoughts from 2004–05 to 2009–10 and 2013 might be because Vietnamese adolescents had improved emotional literacy during this period, which enabled them to recognise and name emotional states. It might also be that higher expectations from parents and teachers for academic success may result in a higher level of stress, and mental health problems and higher likelihood of adolescents having suicidal thoughts. More importantly, as demonstrated in this study, it might also be that adolescents were cumulatively exposed to more forms of victimisation, including child maltreatment, peer or sibling victimisation and the newly emerging forms of victimisation like cyber bullying, which increased their risks of having suicidal ideas.

When compared with results from other low- and lower-middle income countries, the prevalence of suicidal thoughts and plans in our sample is lower than those reported in some countries, but higher than in others. For example, it was reported that 16.3 % of the 13–15-year-old Filipino students who participated in the Global School-based Student Health Survey had seriously considered attempting suicide in the past 12 months, while these were lower for Sri Lanka (9.9 %), Indonesia (4.4 %), Cambodia (5.5 %) and Myanmar (0.7 %) [60–63]. These differences suggest that country-specific characteristics beyond economic development may play an important role in adolescents’ suicidal thoughts and plans. Such characteristics may include adolescents’ risks of exposure to victimisation, as well as available policy, suicide prevention and mental health care programs in each country.

### Gender differences in adolescents’ involvement in risky behaviours, mental health problems and suicidal thoughts and plans

This study replicated results reported previously in Vietnam [17, 40, 48, 64, 65] as well as in other countries [20, 66] about the significantly higher likelihood of boys to engage in externalising behaviours, including smoking, alcohol drinking, physical fighting, carrying a weapon or having multiple sexual partners, and of girls to have internalising problems, including depression and anxiety, or suicidal ideas. In fact, girls in this survey were nearly three times more likely to have suicidal thoughts and two times more likely to have suicidal plans in the previous year than boys (Table 1).

A higher risk of boys engaging in externalising behaviours than girls may be attributable to a higher likelihood of them being affected by peer pressure to be involved in behaviours not permitted by adults [48]; whereas the increased risk of suicidal ideation among girls indicated gender inequality in the Vietnamese society. Despite increased people’s awareness and the Vietnamese government’s effort in addressing this problem, including prohibition of sex-selective abortions and provision of equal opportunity for education for every child, preference towards a son remains in Vietnam, especially in rural areas [67, 68]. The country had a 2014 male:female sex ratio at birth of 112:100 [69]. This is because boys are believed to carry on the family name while girls do not, and sons are expected to care for their parents in old age [70]. At the family level, girls are also expected to do more household chores and they have less opportunities for education [71]. The mean years of schooling for Vietnamese girls are 7.0 while these are 7.9 for boys. At the community level, girls have less opportunities for employment: 82 % of males aged 15 and above participate in the workforce while only 73 % of females do [72]. In addition, Vietnamese girls have been shown to be more likely to experience neglect and emotional abuse than boys [9]. All of these factors may have resulted in a higher likelihood of symptoms of mental health problems and suicidal ideas among girls compared to boys in this setting.

### Directions for future research and implications for practice and policy

This study provides important implications for research, policy and practice in Vietnam. Future research on exposure to violence among Vietnamese adolescents should not be limited to individual forms of violence. Further longitudinal investigations, taking into account frequency and intensity of the victimisation experiences, to facilitate improved understanding of suicidal behaviours among adolescents in Vietnam are needed. Such investigations will help provide a comprehensive evidence base to inform appropriate policies and the development of national programs for mental health care and suicide prevention of children and adolescents in Vietnam.

The Prime Minister of Vietnam’s Decision 1215/QD-TTg in 2011 marked a recent advance in recognition of the needs of people with severe psychotic disorders in the country. It aimed to encourage involvement of families and communities in providing support and functional rehabilitation for these patients and provide funding for different projects in 2011–20. However, the prevalence of non-psychotic mental health problems and suicidal ideation and behaviours remains less well recognised. There is no national program for the care of mental health, prevention of suicidal ideation or behaviours and prevention of violence among children and adolescents in Vietnam. It is clear from these data that such programs should focus on risk-factor reduction, in particular addressing gender inequality and reducing violence towards children and adolescents within families, schools and neighbourhoods as a crucial mechanism to prevent health risk behaviours and improve the mental health of Vietnamese young people.

## Conclusion

Poly-victimisation is associated with involvement in health risk behaviours, poor mental health and increased likelihood of suicidal thoughts and plans among adolescents in Vietnam, but is yet to be recognised. In order to improve the mental health and full social and economic participation of young people, these data suggest that violent victimisation warrants an urgent national response.

## Abbreviations

CI: Confidence Interval
DASS-21: Depression, Anxiety and Stress Scale-21
DASS-21-V: Vietnamese validation of the Depression, Anxiety and Stress Scale-21
JVQ R-2: Juvenile Victimisation Questionnaire Revised 2
SAVY: Survey Assessment of Vietnamese Youth
YRBS: Youth Risk Behaviours Survey
